# Pioneering next-generation bioactive materials for endodontics: Insights of mitochondrial biology

**DOI:** 10.1016/j.bioactmat.2026.05.040

**Published:** 2026-06-04

**Authors:** Shi Cheng, Xin-Ya Liu, Lu Zhou, Han-Qing Mao, Yuan-Hao Wen, Xiang Meng, Lu Zhang, Zhi Chen

**Affiliations:** a State Key Laboratory of Oral & Maxillofacial Reconstruction and Regeneration, Key Laboratory of Oral Biomedicine Ministry of Education, Hubei Key Laboratory of Stomatology, School & Hospital of Stomatology, Wuhan University, Wuhan, 430079, PR China; b Department of Cariology and Endodontics, School and Hospital of Stomatology, Wuhan University, Wuhan, 430079, PR China

**Keywords:** Mitochondria, Endodontics, Vital pulp therapy, Dental pulp regeneration, Dental pulp revascularization, Next-generation materials

## Abstract

Endodontics, a vital discipline in dental medicine, focuses on pulp health preservation, disease treatment, and tissue regeneration, critically dependent on bioactive materials for therapeutic success. Conventional bioactive materials primarily focus on cellular-scale interactions, but with the advent of precision medicine, emerging evidence demonstrates that certain key organelles can influence disease progression by regulating cell fate. Mitochondria, beyond their energy-producing role, are central to cellular signaling, metabolic regulation, and inflammatory responses, profoundly impacting pulp cell survival, differentiation, and adaptation to pathological conditions such as hypoxia or inflammation. Therefore, this leading opinion paper synthesizes cutting-edge insights into mitochondrial biology and their implications for endodontics, proposing a shift in bioactive material design. Firstly, the pivotal roles of mitochondria in core cells relevant to endodontics is delinated. Subsequently, how existing endodontic materials impact mitochondria is described. Afterwards, the latest advances in mitochondria-based bioactive materials for endodontics are analyzed. Finally, the potential to pioneer next-generation endodontic bioactive materials through mitochondrial biology is explored. This paper aims to provide new possibilities for developing more advanced endodontic bioactive materials at the subcellular level.

## Highlights

• This leading opinion paper proposes a paradigm shift from cellular-scale to mitochondrial-scale endodontic material design, providing new possibilities for pioneering next-generation endodontic materials.
• This paper systematically details the critical role of mitochondria in dental pulp cells, uniquely linking organelle biology to clinical endodontic outcomes.
• This paper evaluates the effects of existing endodontic materials on mitochondria, highlighting an underexplored dimension of their biological impact.
• This paper synthesizes cutting-edge advances in mitochondria-targeted materials for endodontics.

## Graphical abstract

**Figure:**
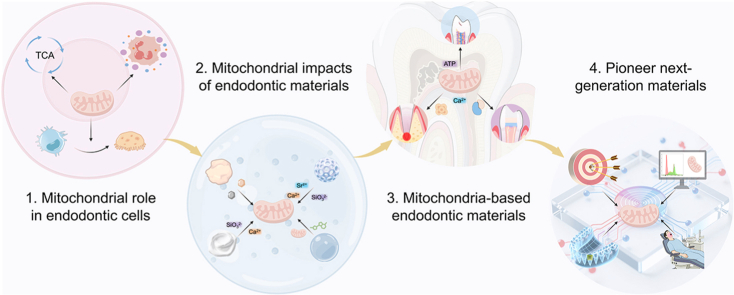

## 1 Introduction

Endodontics, as a critical branch of dental medicine, is dedicated to preserving pulp health, treating pulp and periapical diseases, and exploring the regenerative potential of pulp tissue [1]. Bioactive materials play an indispensable role in this process, from traditional pulp capping agents and root canal filling materials to modern tissue engineering scaffolds [[2], [3], [4]]. However, current bioactive materials still face challenges in biocompatibility, tissue repair and regeneration induction, and long-term efficacy predictability [5]. Conventional bioactive materials primarily target the cellular scale, while their ability to precisely manipulate subcellular structures remains underdeveloped [6,7]. These limitations underscore the necessity to explore regulatory mechanisms at the subcellular level—particularly focusing on mitochondria [8]. In the physiological and pathological processes of pulp and periapical tissues, including the proliferation and differentiation of dental pulp stem cells (DPSCs), odontoblast function maintenance, immune cell responses, and cellular adaptation under pulp inflammation or hypoxia, mitochondrial functional states play a crucial regulatory role [[9], [10], [11]].

**Fig. 1:**
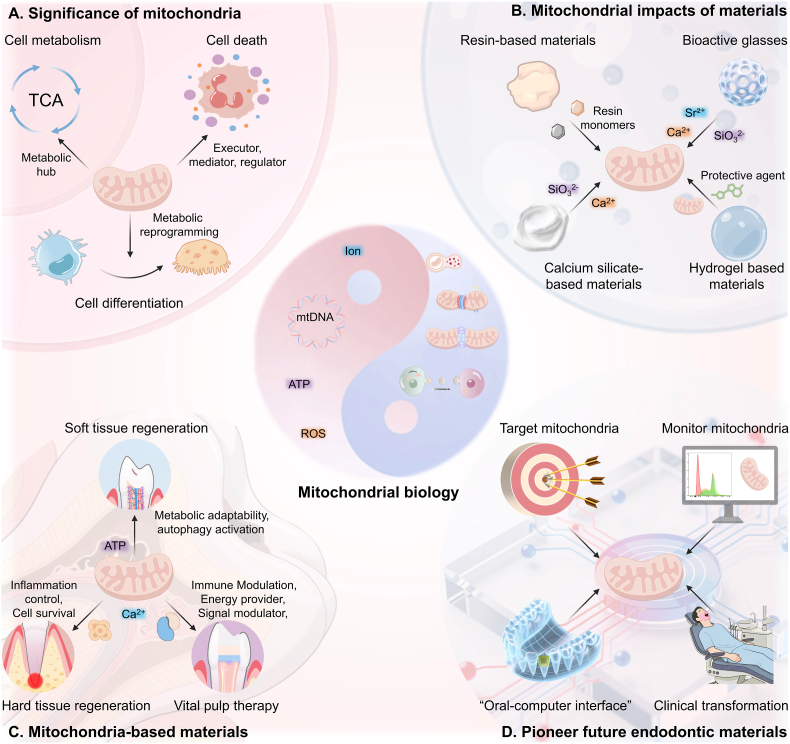
This schematic illustrates the pivotal role of mitochondria in endodontic treatment and the design of next-generation bioactive materials. (A) Mitochondrial regulation in endodontics: Mitochondria orchestrate core processes in dental cells. Their dysfunction disrupts odontoblast activity and pulp homeostasis, influencing repair and regeneration outcomes. (B) Endodontic material-mitochondria interplay: Current materials modulate mitochondrial function via monomer, ion and cargo. (C) Mitochondria-interacting endodontic materials have demonstrated therapeutic potential across vital pulp therapy and tissue regeneration. (D) Future endodontic bioactive materials should focus on four key directions: 1) enhancing mitochondrial targeting; 2) promoting real-time mitochondrial monitoring; 3) developing “oral-machine interfaces” for smart therapeutics, and 4) accelerating clinical translation.

**Fig. 2:**
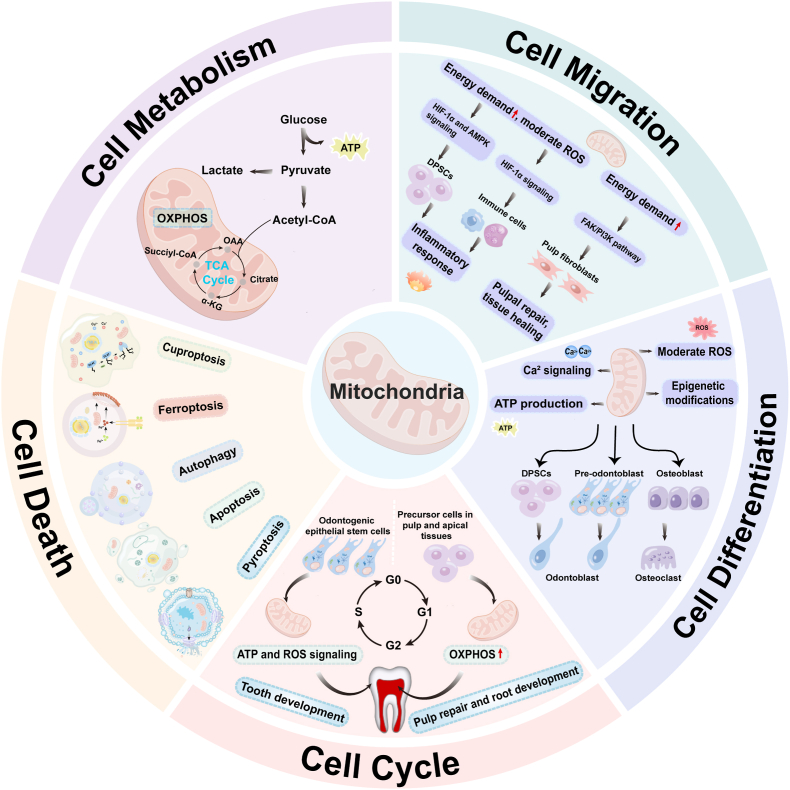
Mitochondrial regulation of cellular fate decisions in endodontic repair and regeneration: established mechanisms, contentious issues, and critical knowledge gaps. (1) Mitochondrial regulation of cell metabolism: Mitochondria orchestrate metabolic homeostasis in dental pulp cells. While the shift from oxidative phosphorylation to glycolysis during early inflammation is well-documented, the precise ATP threshold required for odontogenic differentiation versus fibrotic repair remains undefined, and whether forced metabolic switching can override cell lineage commitments is controversial. (2) Mitochondrial regulation of cell migration in DPSCs, immune cells, and fibroblasts via ROS dynamics and FAK/PI3K pathways. However, direct evidence regarding the impact of mitochondria on the migration of pulp immune cells is currently lacking. (3) Mitochondrial regulation of cell differentiation by metabolic reprogramming, ROS, Ca^2+^ signaling, and epigenetic modifications. (4) Mitochondrial regulation of the cell cycle via energy balance and metabolite flux. (5) Mitochondrial regulation of cell death through mitochondrial outer membrane permeabilization, mtROS, and release of damage-associated molecular pattern (DAMP) release. However, as cell death modalities diversify, which mode predominates in different pulp diseases remains unclear.

**Fig. 3:**
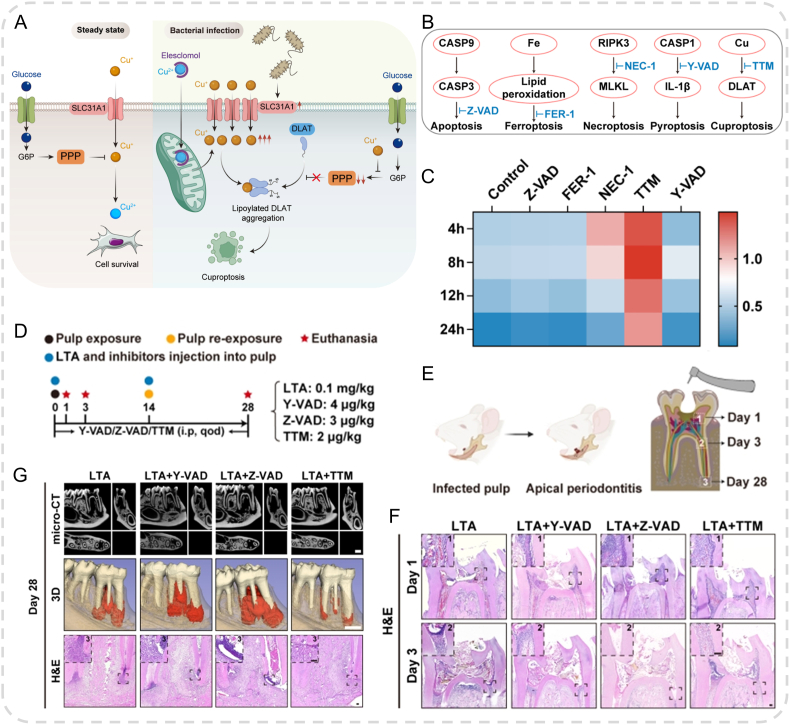
(A) Illustration of bacteria-induced cuproptosis exacerbating pulpitis by suppressing the pentose phosphate pathway. (B) Overview of various cell death mechanisms. (C) Heatmap displaying mDPC6T cell viability following pretreatment with various cell death inhibitors and subsequent exposure to ES-Cu (Copper ionophore). (D) Schematic representation of the rat pulpitis model and treatment regimen. (E) Timeline of pulpitis progression and pulp tissue examination post-induction. (F) H&E staining of rat pulp tissues in the lipoteichoic acid (LTA)-induced pulpitis model, with specific focus on the odontoblast layer at the necrotic front, following treatment with different inhibitors. (G) 2D micro-CT and 3D reconstruction images of periapical tissue sections in the rat LTA-induced pulpitis model, with red regions indicating periapical bone loss [12].

**Fig. 4:**
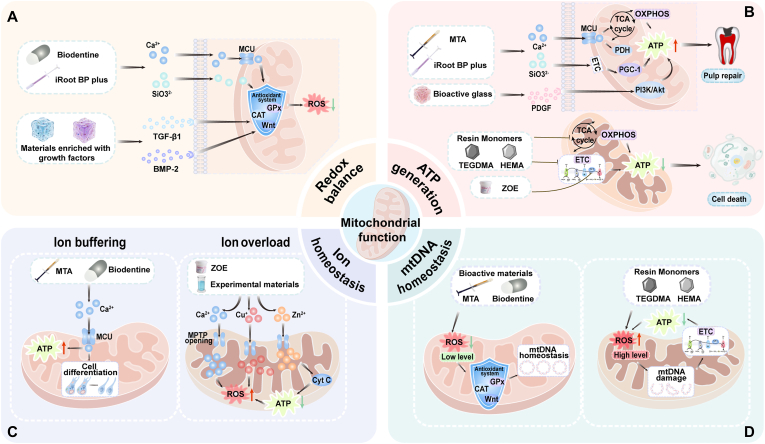
Context-dependent effects of dental materials on mitochondrial function: therapeutic benefits, toxicological risks, and unresolved mechanistic ambiguities. (A) Redox balance: Bioactive materials (e.g., CaSiO_3_ cements). mitigate oxidative stress by scavenging ROS and enhancing antioxidant enzyme activity, while growth factors (e.g., TGF-β1) further protect mitochondria via downstream signaling pathways. However, complete ROS ablation may disrupt physiological redox signaling required for stem cell differentiation. (B) ATP synthesis: Ion-releasing materials (e.g., mineral trioxide aggregate (MTA), Biodentine) boost ATP production by activating oxidative phosphorylation and TCA cycle enzymes, whereas resin monomers (e.g., TEGDMA) or eugenol inhibit electron transport chain complexes, compromising energy metabolism. (C) Ion Homeostasis: CaSiO_3_ materials regulate mitochondrial Ca^2+^ buffering to promote cell differentiation. Nonetheless, the threshold distinguishing physiological matrix synthesis signals from pathological mPTP opening remains unmapped. (D) MtDNA homeostasis: Biocompatible materials preserve mtDNA homeostasis via transient ROS signaling, while oxidative stressors (e.g., resin monomers) induce mtDNA damage, disrupting electron transport chain function and perpetuating metabolic dysfunction. In addition, the hierarchical interaction between these mitochondrial parameters (e.g., whether redox buffering overrides ion homeostasis) and their temporal sequence during the inflammatory-to-reparative transition requires further validation.

**Fig. 5:**
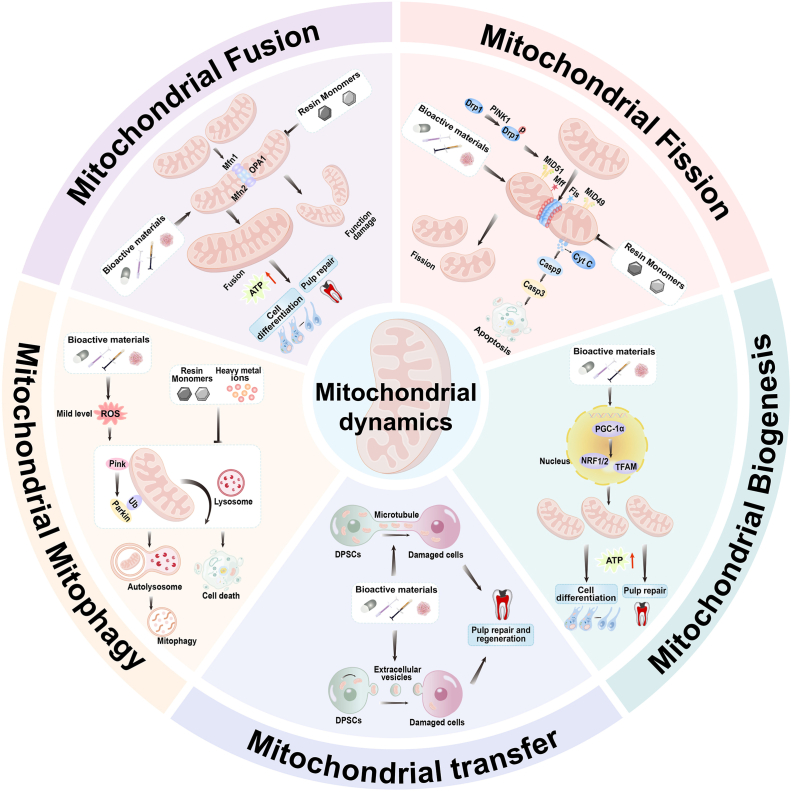
Effects of bioactive materials on mitochondrial dynamics: proposed mechanisms, conflicting interpretations, and empirical uncertainties. (1) Bioactive materials regulate mitochondrial fusion or fission through Mfn1/2, OPA1 or Drp1. Notably, excessive mitochondrial fusion can also induce dysfunction, while moderate fission facilitates clearance of damaged mitochondria; thus, a uniform therapeutic strategy is inappropriate. (2) PGC-1α/NRF1/TFAM-driven mitochondrial biogenesis is enhanced by regenerative materials (e.g., MTA), expanding functional mitochondrial pools to meet energy demands during pulp repair and odontogenic differentiation. (3) Mitochondrial transfer from DPSCs to stressed cells via tunneling nanotubes or exosomes. Although mitochondrial transfer has been demonstrated to exert beneficial effects in pulp diseases, dental materials capable of directly inducing this phenomenon have not been reported. (4) Bioactive material-induced mitophagy (via PINK1/Parkin) likely complements dynamics regulation to eliminate damaged mitochondria.

Leveraging insights from mitochondrial biology, this leading opinion paper aims to explore how these concepts can be applied to endodontics, particularly in guiding the innovation of next-generation bioactive materials. First, this paper elucidates the indispensable role of mitochondria in regulating core biological processes of pulp cells, including metabolism, migration, differentiation, and cell fate decisions (Fig. 1A, 2, 3 and Table 1). Subsequently, the impacts of current endodontic materials on mitochondrial function and dynamics are critically evaluated (Fig. 1B, 4, 5 and Table 2). Following this, the recent advances of mitochondria-interacting bioactive materials are discussed, covering a spectrum from vital pulp therapy to dental pulp regeneration (Fig. 1C, 6-9 and Table 3). Finally, a forward-looking perspective on pioneering next generation endodontic bioactive materials is proposed (Fig. 1D and 10).

**Table 1:** Mitochondrial regulation of key cellular processes in endodontics.

Cellular process	Key mitochondrial roles	Key cells	Ref.
Cell metabolism	Energy hub: ATP production via oxidative phosphorylation Metabolic reprogramming: switch between oxidative phosphorylation and glycolysis	DPSCs, Odontoblasts, SCAP and fibroblasts	[[13], [14], [15], [16], [17]]
Cell migration	Energy supply: ATP for cytoskeleton dynamics Signal transduction: mtROS as signaling molecules Ca^2+^ homeostasis: Regulates signaling pathways	DPSCs, SCAPs, immune cells and fibroblasts	[[18], [19], [20]]
Cell differentiation	Metabolic reprogramming: Shift from glycolysis to oxidative phosphorylation Signal regulation: mtROS activates key pathways (e.g., Wnt/β-catenin) Epigenetic modification: Influences histone and DNA methylation	DPSCs, dental papilla cells, progenitor cell, osteoclast precursor and immune cells	[[21], [22], [23], [24]] [[25], [26], [27], [28]]
Cell cycle	Energy supply: Provides ATP for cell cycle checkpoints Signaling molecules: ROS levels regulate cell cycle progression	DPSCs, macrophages	[29,30]
Cell death (Table S1)	Apoptosis: Releases Cyto C via mitochondrial outer membrane permeabilization to activate caspase cascades Pyroptosis: mtROS/mtDNA activates the NLRP3 inflammasome Ferroptosis: mtROS promotes lipid peroxidation, mitochondria participate in the synthesis of Fe-S clusters Cuproptosis: copper ions bind to lipoylated mitochondrial enzymes (DLAT), triggering proteotoxic stress and aggregation	Multiple cell death modes involving various cell types	[[31], [32], [33], [34], [35], [36], [37], [38], [39], [40]] [12,41,42]

**Table 2:** Impacts of endodontic materials on mitochondria.

Classification	Beneficial Effects & Mechanisms	Detrimental Effects & Thresholds	Ref.
**CaSiO_3_-Based Materials**	**• Redox balance**: ↑Glutathione (GSH)/Glutathione Disulfide (GSSG) ratio via Nrf2-Keap1 axis **• ATP generation**: Ca^2+^-dependent pyruvate dehydrogenase activity↑ **• Ion homeostasis**: mitochondrial calcium uniporter -mediated Ca^2+^ cycling **• Biogenesis**: PGC-1α-mediated TFAM expression↑	• **Dose-dependent toxicity**: Excessive Ca^2+^ may induce mitochondrial permeability transition pore opening	[2,3] [[43], [44], [45], [46], [47], [48]] [49,50]
**Resin Monomers**	/	**• Redox imbalance**: TEGDMA undergoes redox cycling **• ATP generation**: Complex I inhibition **• Mitochondrial Fission**: Drp1↑→excessive fission	[13] [[51], [52], [53], [54], [55], [56]]
**Zinc Oxide Eugenol**	/	**• ATP blockade**: Cytochrome *c* oxidase inhibition **• Ion dysregulation**: Zn^2+^interferes with enzymes →ROS production	[57] [58]
**Bioactive Glasses**	• **Energy substrate**: Si^4+^ ↑glucose uptake **• Redox modulation**: Cu^2+^-dependent SOD mimicry **• Redox imbalance**: reduce oxidative stress	• **Degradation byproducts toxicity**: induce fibrotic reactions	[[59], [60], [61], [62], [63], [64], [65], [66], [67], [68]]
**Growth factors-embedded materials**	**• Redox defense**: TGF-β1 and BMP-2↑HO-1 **• ATP regulation**: PDGF ↑ATP synthase β-subunit	**• Mismatched material degradation fate** **• High production costs**	
**Hydrogels**	**• Intelligent release**: prolonged release of agents **• Sustain functionality of bio-derived materials**	**• Insufficient mechanical strength** **• Suboptimal cross-linking within deep tissue**	[[69], [70], [71], [72], [73]]

**Fig. 6:**
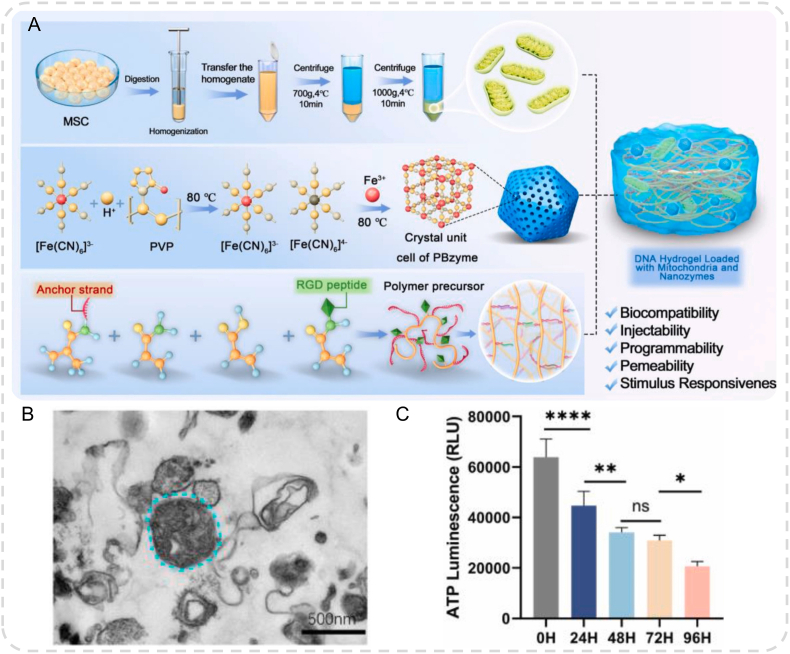
(A) Schematic of the fabrication process of DNA hydrogel loaded with MSC-derived Mitochondria and nanozymes. (B) TEM image of isolated mitochondria. (C) ATP generation assay of isolated mitochondria at various time points [71].

**Fig. 7:**
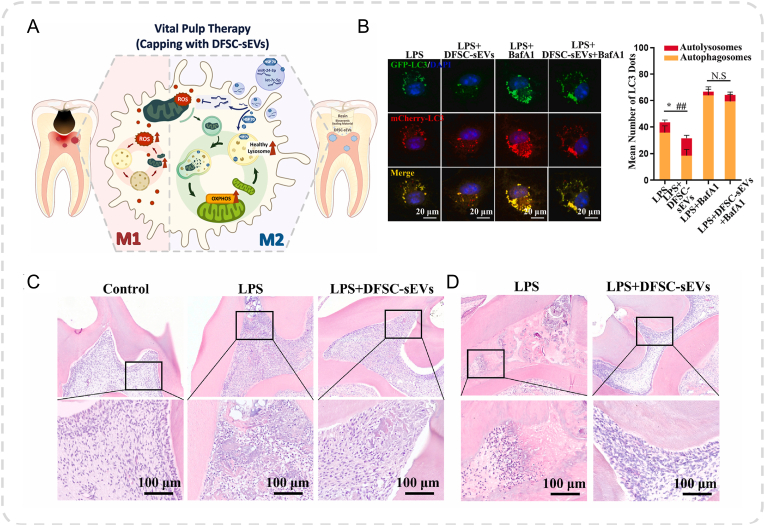
(A) Schematic of DFSC-sEVs ameliorating pulpitis by reprogramming macrophage metabolism. (B) Autophagic flux was measured following exposure to LPS or DFSC-sEVs. The accompanying graph illustrates the quantification of LC3 puncta. (C) H&E staining demonstrated the infiltration of inflammatory cells in LPS-induced pulpitis rat models post-DFSC-sEVs capping. (D) H&E staining revealed pulpal healing in LPS-induced pulpitis rat models following DFSC-sEVs capping [74].

**Fig. 8:**
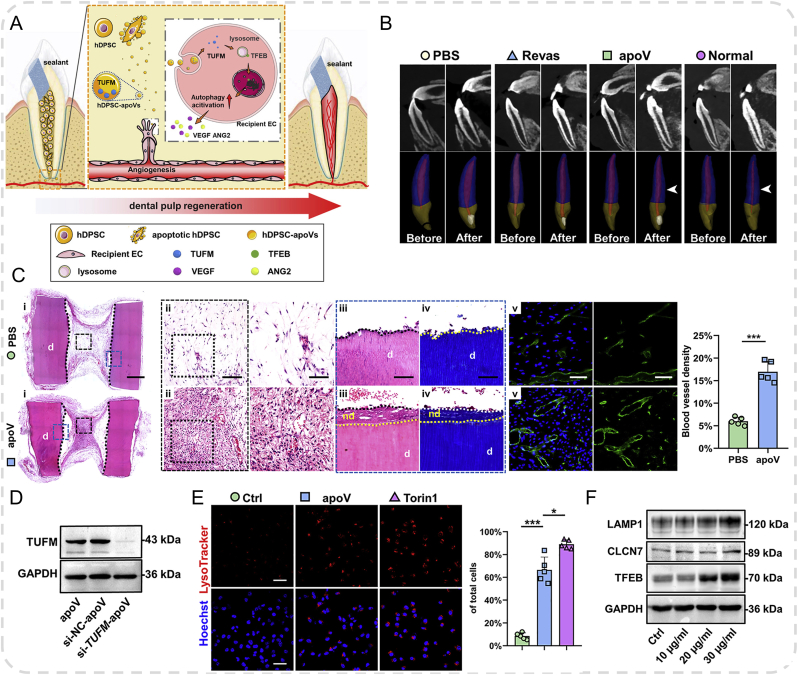
(A) Diagram of hDPSCs promote angiogenesis and dental pulp regeneration through the TFEB-autophagy pathway, mediated by apoV-carried TUFM. (B) CBCT scans (top row) and 3D reconstructions (bottom row) of incisors pre- and post-treatment, revealing enhanced dentin formation (white arrows) in the apoV and control groups. (C) H&E staining, Masson's Trichrome staining and immunofluorescence imaging of sections depict regenerated pulp tissue within tooth scaffolds and corresponding of the same regions. (D) Western blotting analysis indicates the presence of TUFM in apoVs. (E) Representative confocal microscopy images of lysosome in ECs after different treatment. (F) Lysosomal markers (LAMP1, CLCN7) and TFEB levels in ECs were quantified by Western blot following treatment with apoVs at increasing doses [75].

**Fig. 9:**
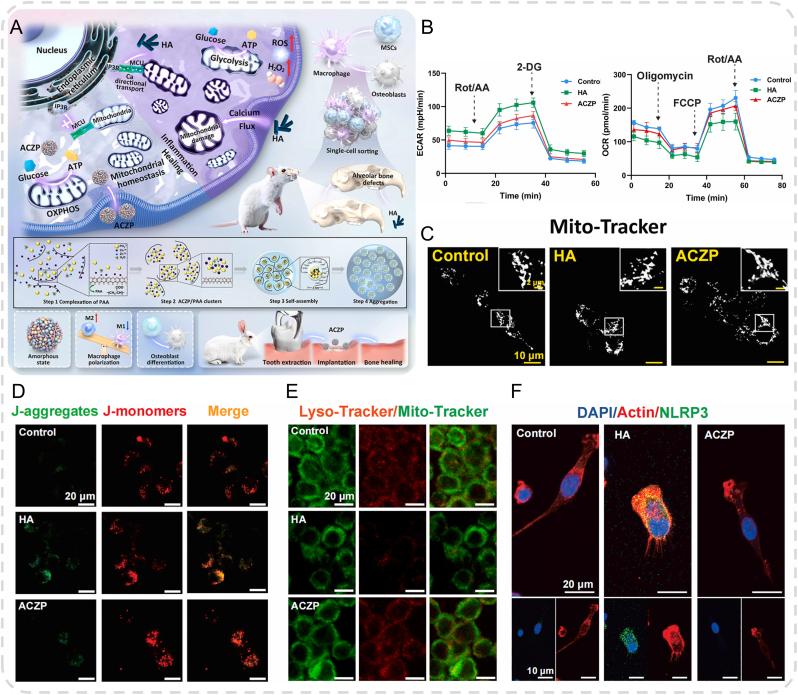
(A) Schematic illustration depicting how ACZP facilitates macrophage-mediated alveolar bone regeneration by regulating energy metabolism and mitochondrial homeostasis. (B-C) Extracellular acidification rate was measured in macrophages with or without HA or ACZP stimulation. Oxygen consumption rate was assessed in macrophages under the same conditions. (D) Macrophage mitochondrial membrane potential changes and morphological analysis were conducted post JC-1 staining with added nanomaterials; green fluorescence indicates JC-1 aggregates, while red fluorescence signifies JC-1 monomers in the cytoplasm. (E) Images showed macrophages stained with Mito-Tracker (green) and Lyso-Tracker (red) to illustrate the co-localization of mitochondria and lysosomes. (F) Representative immunofluorescence images display NLRP3 staining (green) in macrophages treated with various nanomaterials [76].

**Table 3:** Mitochondria-centric therapeutic strategies for endodontic and related diseases.

Application Area	Role of mitochondria	Mitochondria-centric strategy	Ref.
Vital pulp therapy	∙ Activates inflammatory signaling ∙ Mitophagy promotion	∙ Mitochondria-targeted antioxidants ∙ sEVs for triggering mitophagy	[17,34,35] [74,77]
Regenerative endodontic procedures	∙ Mediate SCAP adaptation to hypoxia ∙ TUFM-mediated angiogenesis	∙ apoVs activate TFEB-autophagy pathway ∙ Metabolic reprogramming	[75,[78], [79], [80], [81]]
Periapical bone regeneration	∙ Osteogenesis: dependent on oxidative phosphorylation ∙ Osteoblast oxidative phosphorylation shift ∙ Reprogramming immune cells	∙ Restoring mitochondrial homeostasis ∙ Metabolic switching	[76,82,83]

**Fig. 10:**
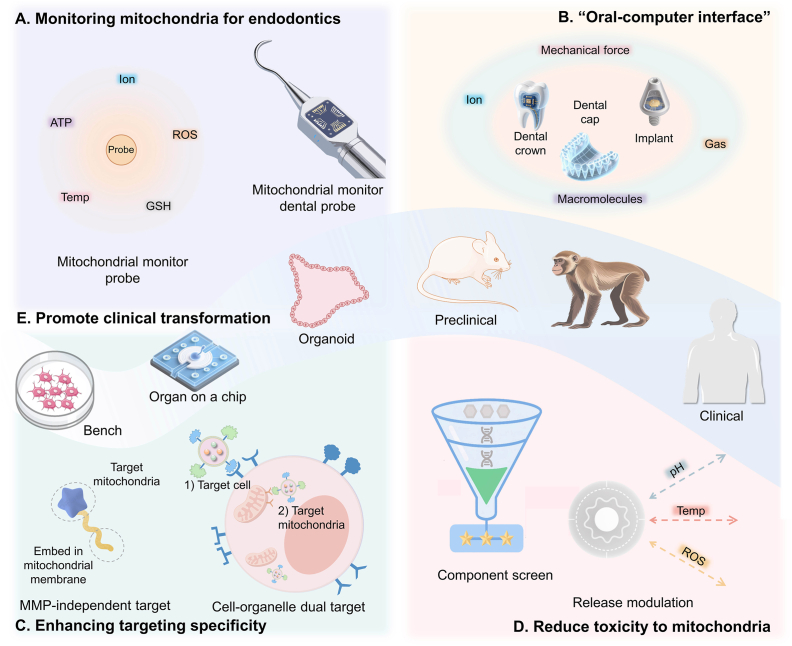
Opinion of pioneering endodontic materials: innovative concepts, technical barriers, and translational uncertainties. (A) Mitochondrial monitoring: Emerging tools such as blinking fluorescent bioprobes enable real-time imaging of mitochondrial biomarkers (ATP, GSH, ROS), offering earlier detection of pulp pathologies than conventional methods. Given the confined workspace and limited optical access typical of endodontic procedures, future probe designs could incorporate miniaturized mitochondrial-monitoring modules. However, fluorescent bioprobes in endodontics face challenges, and the feasibility of intra-pulpal probe delivery without compromising tooth vitality remains unproven. (B) Mitochondrial assessment modalities: The oral cavity's biomarker-rich microenvironment supports non-invasive interfaces (e.g., mechanoluminescent dental splints, glucose sensors) to monitor mitochondrial-linked metabolites (salivary glucose) and biomechanical signals. However, the sensitivity/specificity required to detect localized pulpal oxidative stress from oral fluid biomarkers remains empirically undefined. (C) Precision mitochondrial targeting: mitochondrial membrane potential-independent targeting and bio-recognition strategies (dual cell-organelle targeting) enhance drug delivery specificity to pulp mitochondria, improving treatment efficacy. (D) Mitochondria-safe material design: Next-generation materials should prioritize component and controlled ion release to avoid mitochondrial toxicity. But the dose-response relationship between material ion elution and specific mitochondrial parameters is inadequately mapped for clinical prediction. (E) Promoting clinical transformation: The transition of bioactive materials from laboratory research to clinical application involves a lengthy process. Given the increasing ethical constraints on animal testing, organoids and organ-on-a-chip technologies have emerged as alternatives.

Clinically, to address the need for actionable clarity in mitochondrial-targeted endodontic therapies, we propose four concrete strategic propositions that structure this paper: 1) Mitochondrial functional parameters (such as mtROS) should serve as vital reference metrics for biocompatibility evaluation, as these indicators are more sensitive and precede changes detected by conventional viability assays (e.g., MTT/CCK-8). 2) Current endodontic materials require mandatory screening for mitochondrial toxicity (e.g., electron transport chain inhibition, mtDNA damage) rather than relying solely on cell viability endpoints, recognizing that sublethal mitochondrial injury precedes phenotypic cell death. 3) Moving beyond bio-inertness, future material designs should incorporate mitochondrial modulatory agents (e.g., mitophagy inducers, antioxidant mimetics) to actively support pulp cell bioenergetics and regenerative capacity. 4) Next-generation endodontic materials should integrate chairside mitochondrial monitoring and targeted mitochondrial interventions as therapeutic strategies.

This leading opinion paper systematically analyzes relevant strategies and challenges from the perspective of mitochondria, offering a theoretical foundation and novel insights to steer future directions in the development of mechanism-driven, safer, and more effective endodontic bioactive materials.

## 2 Why do we need to study mitochondrial biology in pulpal and periapical disease?

This section focuses on mitochondrial regulation of critical pulp cell functions (Fig. 2, Table 1), integrating cutting-edge advances to systematically analyze its central role in the pathogenesis, progression, and therapeutic management of endodontic diseases. It explores addresses mitochondrial biology across the spectrum of endodontic disease ranging from pulpal injury and inflammation to periapical bone destruction.

### 2.1 Mitochondrial regulation of the cell metabolism

In dental pulp tissue, specialized cells including odontoblasts, DPSCs, fibroblasts, and vascular endothelial cells critically depend on mitochondrial function to sustain their physiological activities [84]. Odontoblasts are the primary cells responsible for dentin formation, and mitochondria regulate the secretion of dentin matrix proteins (such as dentin sialophosphoprotein, DSPP) via Ca^2+^ homeostasis and ATP production [13]. Mitochondrial dysfunction leads to dentin mineralization defects, making the tooth structure fragile and more susceptible to carious damage. DPSCs are responsible for maintaining the vitality of dental pulp tissue and responding to external stimuli such as injury or microbial infection. When mitochondrial function is impaired, ATP production is reduced and oxidative stress is increased, weakening the repair capability of DPSCs and exacerbating inflammation. Apical papilla stem cells (SCAPs) play an essential role in root development and tissue regeneration. Mitochondria support their proliferation and differentiation by balancing glycolysis and oxidative phosphorylation [14]. Studies indicate that inhibition of mitochondrial oxidative phosphorylation impedes the differentiation of SCAPs into odontoblast or osteoblast lineages, suggesting that metabolic reprogramming is a crucial regulatory node for regenerative potential. Fibroblasts, as the primary mesenchymal cell type in dental pulp tissue, are responsible for synthesizing extracellular matrix, regulating inflammatory responses, and promoting tissue repair [15,16]. When mitochondria are damaged, metabolic dysregulation leads to decreased ATP production and exacerbated oxidative stress, inhibiting fibroblast migration and proliferation. This ultimately weakens the repair potential of dental pulp and may aggravate chronic inflammation or fibrosis [17].

*Opinion*: In preclinical studies, targeted clearance of mitochondrial ROS via MitoTEMPO has been demonstrated in vitro to effectively suppress abnormal proliferation and apoptosis of DPSCs [85]. Furthermore, TFAM promotes the osteogenic differentiation of DPSCs via enhancing oxidative phosphorylation [86]. For gingival fibroblasts, FCCP (a mitochondrial oxidative phosphorylation uncoupler) has been shown to inhibit the anti-inflammatory response they mediate [87]. Therefore, liquid scaffolds (e.g., platelet-rich fibrin) with mitochondrial protectants or metabolic regulators may offer translational promise. However, clinical translation is hindered by the absence of in vivo validation and challenges in maintaining bioactivity and controlled release within scaffolds. Addressing these limitations requires standardized animal model verification and the development of cell-specific targeted delivery systems to ensure efficacy and safety.

### 2.2 Mitochondrial regulation of the cell migration

Cell migration is a key process in response to pulp injury and inflammation, involving active participation from various cell types, including DPSCs and immune cells. The migration of DPSCs is crucial for the formation of tertiary dentin after tooth injury [18]. Yuan et al. found that exosomes derived from SCAPs significantly enhanced the migratory capacity of DPSCs by enhancing mitochondrial fusion-mediated energy metabolism, and mitochondrial inhibition induced by rotenone suppressed the function of exosomes [19]. Besides, activation of mitophagy has been demonstrated to enhance the migratory capacity of human DPSCs [20]. Although direct evidence in endodontic research is currently lacking, mitochondria have been widely proven to regulate the chemotaxis and migration of immune cells through ROS signaling and ATP production [88]. Impaired mitochondrial function may result in the inability of immune cells to migrate efficiently to the site of injury, thereby slowing down the inflammatory response and the repair process. While absolute mtROS quantification would provide definitive thresholds, current technical constraints—including probe off-target effects and sub-mitochondrial compartmentalization—limit reliable measurements to relative fold changes versus controls. Future standardization will likely require target-specific redox sensors monitoring individual protein oxidation states rather than bulk ROS, potentially enabled by advanced ratiometric probes or single-molecule localization microscopy.

*Opinion*: Given that the migration ability of DPSCs is significantly inhibited under stimulation with high concentrations (10 μg/mL) of lipopolysaccharides (LPS), this may significantly suppress tertiary dentin formation [89]. Therefore, in the future, adding components such as mitophagy agonists to pulp capping materials could be a promising strategy for dentin repair in cases of infected pulp exposure.

### 2.3 Mitochondrial regulation of the cell differentiation

In endodontics, cell differentiation is a crucial determinant of pulp tissue repair and regeneration, a process critically dependent on mitochondrial function [90]. Mitochondria influence the differentiation of DPSCs, dental papilla cells, progenitor cells, and osteoclast precursors by modulating metabolic reprogramming, ROS signaling, Ca^2+^ homeostasis, and epigenetic modifications [21]. Mitochondrial oxidative phosphorylation serves as the central driving force for DPSCs differentiation, characterized by elevated mitochondrial membrane potential and enhanced ATP production rate occur, accompanied by activation of the PGC-1α/TFAM axis to promote mitochondrial biogenesis [22]. Notably, moderate mitochondrial ROS elevation activates Wnt/β-catenin signaling to upregulate odontoblastic differentiation markers (DMP1, DSPP) [23,24]. Recent studies demonstrate that mitochondria-targeted probes effectively elevate DSPP and Ca^2+^ levels through membrane potential modulation, thereby promoting osteogenic differentiation [11]. SCAPs, which play a vital role in dentin formation, differentiate into mature odontoblasts during dentinogenesis [91]. Mitochondria support this process by regulating mitochondrial membrane potential, ATP production, and the activity of Complex III and citrate synthase [25]. In the pulp inflammatory microenvironment, the mitochondrial metabolic state of immune cells (such as macrophages, neutrophils and T cells) directly regulates their functional phenotypes [[26], [27], [28]].

*Opinion*: Mitochondrial regulation of cell differentiation is pivotal to pulp regeneration. Targeting the PGC-1α/TFAM axis could enhance the odontogenic differentiation of DPSCs (e.g., via DSPP/DMP1 upregulation) while mitigating oxidative damage. For immune cells, in addition to the extensively studied metabolic modulators and antioxidants, a new direction involves promoting macrophage anti-inflammatory differentiation through mitochondrial function via post-translational modifications, such as lactylation [26]. However, the high diffusivity and pH-lowering effects of lactic acid limit its further application. Replacing lactic acid with sodium lactate and further preparing sodium lactate into nanocrystals loaded onto hydrogels, bioceramics, or pastes may enhance its efficacy in endodontics.

### 2.4 Mitochondrial regulation of the cell cycle

Mitochondria serve as central regulators of the cell cycle in DPSCs and immune cells. For example, Bartold's group showed that differential expression of mitochondrial proteins (8% of total differentially expressed proteins) among DPSCs, periodontal ligament stem cells, and bone marrow mesenchymal stem cells drives lineage-specific cell cycle progression by modulating energy metabolism (ATP) and metabolite flux [29]. Key metabolites such as succinate influence critical checkpoints: 1) G1/S Transition: Succinate dehydrogenase activity modulates HIF-1α signaling, which can indirectly influence cyclin D/E expression via inflammatory pathways. 2) Mitotic Entry: Mitochondrial citrate export fuels cytosolic acetyl-CoA production, promoting histone acetylation (e.g., H3K27ac) that regulates genes involved in mitotic entry. Furthermore, mitochondrial transfer directly mediates the cell cycle: DPSCs transfer functional mitochondria to macrophages via tunneling nanotubes under inflammatory conditions. This process reprograms macrophage cell cycle: reprogrammed immunometabolism promotes a shift from pro-inflammatory M1 macrophages (associated with a proliferative state) towards reparative M2 macrophages, which are characterized by G0/G1 arrest and exit from active cycling [30].

***Opinion***: Rather than relying solely on the passive bioactivity of mineral trioxide aggregate (MTA) or calcium silicate-based cements, we propose functionalizing these materials with metabolic modulators to actively correct succinate-driven checkpoint dysregulation in the pulp injury. For direct pulp capping or partial pulpotomy, incorporating dichloroacetate or controlled-release metformin into the bioceramic matrix could locally reverse the metabolic shift and HIF-1α accumulation characteristic of pulpitis. In regenerative endodontic procedures (RET), isolated DPSC-derived mitochondria could be suspended in autologous platelet-rich fibrin or injectable hydrogels, inducing M2 polarization and G0/G1 arrest that establishes a quiescent, pro-repair niche within the canal. In general, our aim is to enhance the performance of existing materials by coordinating the cell cycle with a reparative microenvironment through pharmacological means, without interfering with standard clinical operating procedures.

### 2.5 Mitochondrial regulation of the cell death

Mitochondria serve as central regulators of cell death, modulating diverse death modalities by integrating signals related to energy metabolism, redox signaling, and membrane permeability [[92], [93], [94]]. Within dental pulp tissue, beyond classical apoptosis and necrosis, cell death manifests in various forms, including non-canonical regulated cell death mechanisms such as ferroptosis, cuproptosis, and panoptosis (Table S1) [95]. Mitochondria dictate the initiation of apoptosis by modulating energy metabolism, ROS levels, and crucial apoptotic signaling pathways, including mitochondrial outer membrane permeabilization [31,32]. In pulpitis, stimuli such as LPS or hypoxia can activate Bax/Bak proteins, inducing mitochondrial outer membrane permeabilization and the subsequent release of cytochrome c into the cytosol. Necrosis is characterized by excessive ROS accumulation, which leads to mitochondrial dysfunction. This dysfunction manifests as mitochondrial membrane potential collapse, ATP depletion, and opening of the mitochondrial permeability transition pore, leading to the loss of plasma membrane integrity and the release of cellular contents (e.g., high mobility group box 1), ultimately inducing necrotic cell death. Pyroptosis is a highly pro-inflammatory form of programmed cell death mediated by inflammatory caspases (primarily caspase-1, -4, -5, and -11) [33]. Mitochondria exert multifaceted crucial functions within the pyroptosis regulatory network: 1) source of ROS; 2) release of DAMPs: released mtDNA can be sensed by inflammasomes, thereby triggering pyroptosis; 3) maintenance of energy supply: mitochondria also provide ATP for the assembly and function of inflammasomes. In pulpitis and periapical periodontitis, pathogen-associated molecular patterns (PAMPs; e.g., LPS) derived from bacteria or host-derived DAMPs (e.g., extracellular ATP) can activate pyroptosis in fibroblasts and infiltrating immune cells (such as macrophages) [34,35]. Neutrophil extracellular trap formation (NETosis) is a programmed cell death pathway specific to neutrophils [[36], [37], [38]]. MtROS, acting in concert with ROS generated by NADPH oxidase (NOX), is essential for the activation of downstream signaling pathways, such as driving protease translocation into the nucleus and promoting chromatin decondensation [39,40].

Dysregulation of transition metal homeostasis can induce novel forms of cell death, the execution of which depends on mitochondrial lipid peroxidation, collapse, and aggregation of respiratory chain copper toxic proteins [96]. Ferroptosis is an iron-dependent form of programmed cell death mediated by lipid peroxidation [97,98]. Mitochondrial dysfunction increases ROS production, which promotes ferroptosis through lipid peroxidation [41]. Furthermore, iron metabolism and mitochondrial function are interconnected; mitochondria participate in the synthesis of Fe-S clusters, which serve as cofactors for numerous mitochondrial enzymes. Ferroptosis inhibitor Ferrostatin-1 (Fer-1) could alleviate bone loss in apical periodontitis by suppressing lipid peroxidation and inhibiting osteoclast activation [42]. Furthermore, engineered bioactive chitosan-based nanoparticles can inhibit ferroptosis by upregulating antioxidant proteins to scavenge ROS [99]. Cuproptosis is characterized by elevated intracellular copper ion concentrations, which directly bind to lipoylated components within mitochondria [[100], [101], [102]]. Zhang's group discovered that copper ions played a significant role in the initiation and progression of bacteria-induced pulpitis through mitochondria-mediated cuproptosis (Fig. 3A) [12]. They demonstrated that cuproptosis can significantly impact the survival rate of mouse dental papilla cell line 6T (mDPC6T) cells (Fig. 3B and C). Subsequently, they found that the cuproptosis inhibitor could markedly suppress the early and late progression of pulpitis (Fig. 3D–F).

***Opinion***: Given the diversity of cell death modalities and the continued identification of novel forms, it is necessary to distinguish the predominant modes of cell death via single-cell RNA sequencing. Additionally, it should be noted that the core cell death mechanisms may vary at different stages of disease progression [103]. Future pulp capping or root canal irrigation materials incorporating cell death inhibitors may lead to new breakthroughs in endodontics. From a clinical perspective, although inhibitors targeting multiple cell death have been identified and demonstrated to be effective, their small-molecule properties result in short circulation times and potential off-target side effects. Designing these small molecules as prodrugs with stimuli-responsive release mechanisms will facilitate their smart application in clinical settings [104].

In vivo, dental pulp cells rarely encounter isolated pathological stimuli; instead, they confront complex microenvironments in which hypoxia, inflammation, bacterial infection, and metabolic disturbances coexist. These stressors exert synergistic effects on mitochondrial function, causing exponential rather than linear cellular damage. For instance, hypoxia in pulpitis stabilizes HIF-1α, synergizing with NF-κB to drive mtROS production and NLRP3 inflammasome; Hyperglycemia exacerbates LPS-induced mitochondrial injury via oxidative phosphorylation impairment and mtDNA leakage, activating cGAS-STING and type I interferon responses; Concurrently, acidic bacterial metabolites dissipate mitochondrial membrane potential, perpetuating bioenergetic failure. These interactions position mitochondria as critical integrators of environmental stress, suggesting that effective endodontic interventions must target multiple converging pathways—such as combined antioxidant, anti-inflammatory, and pro-biogenic strategies—rather than addressing single insults in isolation. Therefore, future bioactive materials should be engineered to modulate these synergistic mitochondrial responses within the complex pathological context of the dental pulp.

Given the central role of mitochondria in pulp pathophysiology and repair outlined above, understanding how clinical materials modulate mitochondria becomes essential. The following section examines the specific impacts of current endodontic biomaterials on mitochondrial function and dynamics.

## 3 What are the impacts of existing endodontic materials on mitochondria?

This section provides a comprehensive summary of the effects of bioactive materials on mitochondria, analyzing from two distinct perspectives: mitochondrial biological processes (Fig. 4, Fig. 5) and material compositional characteristics (Table 2).

### 3.1 CaSiO_3_-based materials

CaSiO_3_-based cements—including MTA, Biodentine, and iRoot BP Plus—are widely used in endodontics. A defining feature of these materials is the sustained release of bioactive ions, particularly Ca^2+^ and SiO_3_^2−^, which play pivotal roles in modulating cellular responses via mitochondrial pathways.

#### 3.1.1 Effect on redox balance

Studies show that extracts from Biodentine can reduce intracellular ROS levels in DPSCs, while simultaneously enhancing the activity of endogenous antioxidant enzymes such as superoxide dismutase and catalase [2,43]. The released silicate and Ca^2+^ can activate intracellular antioxidant signaling cascades, including the Nrf2 pathway, thereby protecting mitochondria from oxidative damage [3,[44], [45], [46], [47], [48]]. This redox-stabilizing effect contributes to reduced inflammation and supports cell survival under stress conditions [49,50].

#### 3.1.2 Effect on ATP generation

CaSiO_3_ materials enhance mitochondrial bioenergetics by promoting oxidative phosphorylation [105]. Specifically, the influx of extracellular Ca^2+^ into cells is taken up by mitochondria via the mitochondrial Ca uniporter (MCU) , leading to activation of pyruvate dehydrogenase and key enzymes in the TCA cycle. This metabolic shift increases NADH production and boosts electron transport chain efficiency, resulting in elevated ATP synthesis. Clinical studies following direct pulp capping with iRoot BP Plus revealed increased cristae density in odontoblast mitochondria and enhanced activity of isocitrate dehydrogenase (a rate-limiting TCA cycle enzyme) within seven days of treatment, underscoring the material's role in stimulating mitochondrial metabolism [48].

#### 3.1.3 Effect on ion homeostasis

Mitochondrial ion buffering is a crucial mechanism for maintaining cytosolic Ca^2+^ homeostasis during signaling events related to differentiation and repair [106,107]. The gradual release of Ca^2+^ from CaSiO_3_ cements creates a localized ionic microenvironment that promotes transient elevations in intracellular Ca^2+^, acting as a physiological signal for DPSC differentiation and dentinogenesis [108,109]. However, prolonged or excessive Ca^2+^ elevation—though not typically observed with clinically applied doses—could theoretically trigger mPTP opening and initiate apoptosis [110].

#### 3.1.4 Effect on mitochondrial dynamics

CaSiO_3_ materials positively influence mitochondrial dynamics. Specifically, these materials promote mitochondrial biogenesis by activating the PGC-1α signaling pathway, increasing both mitochondrial mass and functional capacity in pulp cells [3]. Upregulation of fusion proteins such as Mfn1/2 and OPA1 has also been observed, favoring the formation of an interconnected mitochondrial network associated with improved metabolic efficiency and stress resistance. In addition, iron oxide nanoparticles have been demonstrated to alleviate diseases by enhancing mitochondrial transfer in mesenchymal stem cells [111]. In the future, materials capable of promoting mitochondrial transfer may be utilized for the treatment of endodontic diseases.

### 3.2 Resin-based materials

Resin-based sealers and restorative materials, commonly containing monomers such as triethylene glycol dimethacrylate (TEGDMA) and 2-hydroxyethyl methacrylate (HEMA), are widely used in endodontics [112,113]. However, these hydrophilic monomers can leach from polymerized resins and accumulate in tissues, where they exert significant mitochondrial toxicity.

#### 3.2.1 Effect on ATP depletion

Leached monomers directly impair mitochondrial energy production. TEGDMA and HEMA disrupt the electron transport chain, particularly inhibiting Complex I and III activities, leading to a sharp decline in ATP levels [[51], [52], [53], [54]]. Furthermore, these compounds act as mild uncouplers of oxidative phosphorylation, dissipating the proton gradient across the inner mitochondrial membrane without generating ATP.

#### 3.2.2 Effect on oxidative stress and mtDNA damage

One of the most detrimental effects of resin monomers is the induction of severe oxidative stress. Elevated ROS levels cause oxidative lesions in mitochondrial DNA (mtDNA), including base modifications and single- and double-strand breaks [55]. Given the limited repair capacity of mtDNA, persistent damage impairs transcription of electron transport chain subunits, creating a vicious cycle of further ROS generation and progressive mitochondrial dysfunction [13,56]. This genomic instability is increasingly recognized as a key contributor to long-term cytotoxicity and inflammatory responses.

#### 3.2.3 Effect on dysregulated mitochondrial fission

Exposure to TEGDMA or HEMA results in Drp1 activation and translocation to mitochondria, driving uncontrolled fragmentation of the mitochondrial network [53]. This morphological shift is associated with loss of membrane potential, reduced ATP output, and increased release of pro-apoptotic factors such as cytochrome *c*. Fragmented mitochondria are more susceptible to mitophagy, but chronic exposure may exceed clearance capacity, leading to apoptotic or necrotic cell death. Therefore, dysregulated fission serves as a hallmark of resin-induced mitochondrial distress and a sensitive indicator of early cytotoxicity.

### 3.3 Zinc oxide eugenol

Zinc oxide eugenol (ZOE) has historically been employed as a temporary filling material and intracanal medicament due to its antibacterial properties and soothing effect on irritated pulps. However, its application is now constrained due to well-documented adverse effects on mitochondrial function [114].

#### 3.3.1 Effect on ATP depletion

Eugenol, the organic component of ZOE, acts as a potent inhibitor of mitochondrial respiration. It specifically targets cytochrome *c* oxidase (Complex IV of the electron transport chain), blocking electron flow and halting ATP synthesis [57]. This inhibition leads to rapid depletion of cellular ATP reserves, compromising vital cellular functions and ultimately resulting in necrosis.

#### 3.3.2 Effect on oxidative stress and ion homeostasis

In addition to eugenol's effects, the inorganic component, zinc oxide, releases Zn^2+^ ions that accumulate in mitochondria. At high concentrations, Zn^2+^ interferes with multiple mitochondrial enzymes and induces ROS production, exacerbating oxidative stress [58]. Excessive zinc uptake disrupts Ca^2+^ homeostasis and may synergize with eugenol to amplify mitochondrial dysfunction. Hence, while ZOE offers symptomatic relief, its mitochondrial liabilities warrant caution in clinical decision-making.

### 3.4 Bioactive glasses and ceramics

Next-generation bioactive ceramics and glass-based composites represent a promising frontier in endodontic bioactive material design. These materials are engineered not only for structural support but also to actively engage with biological systems at the metabolic level. Bioactive glasses degrade in physiological environments to release ions such as Si^4+^, Sr^2+^, and Ca^2+^, all of which significantly affect ion homeostasis and enhance mitochondrial activity. Si^4+^ stimulate glucose uptake and glycolytic flux, providing substrates for the TCA cycle and boosting oxidative phosphorylation efficiency [59,60]. When strontium is incorporated into hydrogels, these composites create a favorable niche for pulp regeneration by enhancing ATP production and reducing oxidative stress, thereby improving cell viability and function [61]. However, it should be noted that the accumulation of degradation byproducts from these materials may lead to excessive Si^4+^ levels, potentially inducing fibrotic reactions [62,63].

### 3.5 Growth factor-embedded materials

To bridge the gap between passive scaffolding and active tissue engineering, several next-generation endodontic systems incorporate growth factors designed to guide cellular behavior. Transforming growth factor-beta 1 (TGF-β1), bone morphogenetic protein-2 (BMP-2), and platelet-derived growth factor (PDGF) are among the most studied.

TGF-β1 and BMP-2 activate downstream Smad and non-Smad signaling pathways that upregulate antioxidant defenses, including heme oxygenase-1 (HO-1) and glutathione peroxidase, thereby shielding mitochondria from oxidative injury [[64], [65], [66]]. TGF-β1 has been shown to upregulate the expression of intracellular antioxidant enzymes and mitigate oxidative stress-induced mitochondrial dysfunction [66].

PDGF exerts powerful pro-metabolic effects via the PI3K/Akt/mTORC1 axis, upregulating the expression of TFAM and ATP synthase subunit alpha (ATP5A) [67,68]. In DPSCs, PDGF treatment doubles ATP production rates, accelerating dentin bridge formation and functional recovery after injury. However, the clinical translation of these materials face several challenges, including mismatched degradation rates and factor release kinetics, compromised stability and bioactivity of loaded factors, and high production costs.

### 3.6 Hydrogels

Hydrogels, characterized by their high water retention, excellent biocompatibility, and tunable mechanical attributes, have shown significant promise in endodontics [69]. These materials facilitate sustained release of therapeutic agents, while providing a supportive microenvironment for cell growth and tissue restoration.

#### 3.6.1 Effect on mitochondrial quality maintenance

Variations in the external microenvironment, such as pH levels, ions, redox status, and nutrients, all impact mitochondrial quality [70]. Consequently, developing bioactive materials capable of sustaining mitochondrial quality presents a significant challenge, as mitochondria extracted from cells rapidly become inactive. Therefore, Wang et al. developed a two-pronged approach using a DNA hydrogel modified with polymers to co-deliver nanozymes and viable mitochondria (Fig. 6 A) [71]. Owing to the protective effect of the hydrogels, mitochondrial activitywas maintained with minimal decay over time, sustaining viability for up to 96 hours (Fig. 6B and C). Similarly, Städle's team encapsulated isolated mitochondria in a gelatin-based hydrogel [72]. The initial ATP production was comparable to that of mitochondria in solution, and it sustained ATP generation for up to 24 hours, providing energy support for artificial cells. These results demonstrate that hydrogels not only possess good biocompatibility but also show promise in maintaining mitochondrial quality at the subcellular level.

#### 3.6.2 Delivery of mitochondrial protective agents

Advanced hydrogels exhibit smart responsiveness and functionality in response to a range of environmental cues [115]. Wei's group developed a ROS-responsive hydrogel (SA-RhB@sEVs), integrating sodium alginate (SA) with a ROS-sensitive probe (RhB-AC) and dental follicle stem cell (DFSC)-small extracellular vesicles (sEVs) [73]. This system exhibited controlled release of sEVs in high-ROS environments, ensuring localized and sustained therapeutic delivery. DFSC-sEVs restored the oxidative/antioxidative balance in DPSC mitochondria, demonstrating a comparable effectiveness to the mitochondrial-targeted antioxidant Mito-Tempo in alleviating mitochondrial dysfunction.

### 3.7 Root canal irrigants

#### 3.7.1 Sodium hypochlorite

Sodium hypochlorite (NaOCl), widely employed as a root canal irrigant, exerts concentration-dependent biphasic effects on mitochondrial integrity that critically dictate cellular fate. In DPSCs, concentrations below 1% preserve tubular mitochondrial architectures and metabolic homeostasis, whereas exposure to ≥3% induces severe organelle fragmentation, mitophagy activation, and energetic depletion, thereby compromising osteogenic differentiation potential [116]. This high-concentration–induced mitochondrial damage activates autophagic pathways, compromising the energetic capacity necessary for subsequent osteogenic differentiation and cellular viability. Similarly, in dermal fibroblasts, NaOCl causes irreversible loss of mitochondrial dehydrogenase activity and cell death when concentrations ≥0.05% [117]. These cytotoxic effects are partially attenuated by fetal calf serum supplementation, though mitochondrial dysfunction at lethal concentrations remains refractory to such protection. However, current cytotoxicity assessments predominantly rely on mitochondrial function-based assays such as MTT and XTT, which present significant interpretive limitations: elevated absorbance may reflect metabolic stress responses rather than genuine proliferation, and early apoptotic cells retain residual succinate dehydrogenase activity, potentially generating false-positive viability signals. Consequently, reliance solely on mitochondrial metabolism markers risks conflating adaptive responses with toxicity, necessitating multi-parametric approaches incorporating membrane integrity, morphological, and apoptotic markers to accurately delineate NaOCl-induced mitochondrial dysfunction and ensure reliable translational relevance for endodontic regenerative procedures.

#### 3.7.2 Chlorhexidine

Chlorhexidine (CHX) demonstrates superior efficacy, inducing 25% mitochondrial inhibition at as low as 0.001% and impairing osteogenic differentiation and matrix mineralization in human periodontal ligament fibroblasts at sub-toxic concentrations (0.00012%–0.012%) without affecting cellular proliferation [118]. In odontoblast-like MDPC-23 cells, CHX elicits progressive mitochondrial toxicity through succinate dehydrogenase (SDH) inhibition, with metabolic activity declining 61%–70% across 0.06%–2.0% concentrations in a time-dependent manner, where prolonged exposure substantially exacerbates cytotoxicity compared to brief contact [119]. These findings underscore a critical translational concern: extrusion of these irrigants beyond the apical foramen may irreversibly compromise periapical tissue regeneration through mitochondrial disruption, even at concentrations that spare immediate cell death. However, exclusive dependence on mitochondrial function assays cannot distinguish between transient metabolic suppression and irreversible mitochondrial damage, nor do they account for the dissociation between short-term viability and long-term regenerative capacity evident in CHX-induced differentiation failure.

### 3.8 Calcium hydroxide

Calcium hydroxide (CH), widely employed as an intracanal medicament and pulp-capping agent, yields contradictory evidence regarding its impact on mitochondrial integrity, which warrants critical examination. While initial MTT-based assessments utilizing fibroblastic (L929) and osteoblastic (MC3T3) cell lines suggest CH formulations preserve mitochondrial dehydrogenase activity and metabolic function over extended periods, contrasting data demonstrate severe cytotoxicity in dental pulp fibroblasts at 1 mg/mL concentrations, reducing cell viability to approximately 11% through high pH-mediated disruption of mitochondrial enzymes [120]. This apparent discrepancy likely reflects concentration-dependent thresholds and cell-specific sensitivities obscured by methodological inconsistencies across studies. Furthermore, although CH-induced hydroxyl ion release triggers ROS generation and NF-κB activation pathways intrinsically linked to mitochondrial redox regulation and oxidative phosphorylation dysfunction, the current literature conspicuously lacks direct morphological or functional mitochondrial analyses, relying instead on indirect MTT reduction assays that cannot distinguish between transient metabolic suppression and irreversible organelle damage [121]. Consequently, the assumption that CH exhibits favorable biocompatibility based solely on dehydrogenase activity preservation may be misleading, as prolonged inflammatory signaling and ROS accumulation likely induce mitochondrial oxidative stress unrecognized by conventional viability endpoints. Therefore, comprehensive evaluation of mitochondrial membrane potential, respiratory chain function, and morphological dynamics is essential to accurately characterize CH's bioenergetic impact on periapical and pulpal tissues.

***Opinion:*** Traditional Ca_3_SiO_5_-based materials may induce mitochondria damage due to the rapid release of Ca^2+^. Composite materials consisting of Ca_3_SiO_5_ (core) and Ca_2_SiO_4_ (shell) can be developed, exploiting the stability and sustained Ca^2+^-release properties of Ca_2_SiO_4_ materials [122]. In addition, mesoporous calcium silicate nanomaterials exhibit superior biocompatibility, bioactivity, and notably drug-loading capacity, facilitating smarter applications [123].

Despite their inherent mitochondrial toxicity, resin-based materials offer distinct clinical advantages, including ease of manipulation, superior sealing capacity, and minimal interfacial microleakage. Future optimization could involve incorporating mitochondrial-compatible components to enhance their biocompatibility and clinical applicability.

To circumvent the high cost and poor stability of recombinant growth factors, we propose utilizing small-molecule modulators or autologous extracellular vesicles derived from patient cells as functional supplements to reduce costs while synergistically enhancing endogenous repair factor expression. Notably, the clinical utility of exosomes remains constrained by their low yield. Intracellular vesicles, which demonstrate approximately 16-fold higher yields and enhanced bioactivity compared to exosomes, may emerge as a superior alternative for future clinical applications [124].

Hydrogels with good biocompatibility and intelligence are promising materials in the field of endodontics. Smart hydrogels can dynamically interface with the pathological microenvironment to deliver mitochondrial protectants and even facilitate the transfer of healthy mitochondrial units to rescue stressed cells. More significantly, a variety of bio-derived materials rely on the in vivo-like environment provided by hydrogels to sustain their vitality and functionality, and these bio-derived materials are more amenable to clinical translation. However, hydrogel materials inherently face the issue of insufficient mechanical strength for endodontic applications. Simultaneously, achieving effective cross-linking of hydrogels deep within dental tissue structures remains a challenge.

Building upon these mechanistic insights into material-mitochondria interactions, emerging strategies now aim to harness or modulate mitochondrial function using bioactive materials. The following section reviews recent progress in mitochondria-interacting endodontic materials and future therapeutic directions.

## 4 Advances and perspectives of mitochondria-interacting endodontic materials

This section will focus on the treatment of endodontic diseases and therapeutic goals, systematically exploring strategies for modulating mitochondrial function and the challenges faced (Table 3).

### 4.1 Promoting vital pulp therapy

Vital pulp therapy (VPT) aims to remove the infected or irritated portion of the dental pulp while preserving the vitality and function of the remaining healthy pulp, representing a crucial trend in endodontics [[125], [126], [127], [128]]. Mitochondrial dysfunction, particularly the excessive production of mtROS and release of mtDNA, activates inflammatory signaling pathways (e.g., the NLRP3 inflammasome), exacerbating inflammatory damage and leading to pulp cell death [17,35]. Applying bioactive materials capable of stabilizing mitochondrial function holds promise for alleviating pulpal inflammation and creating a favorable environment for pulp survival [34,77]. For example, Wei's group applied sEVs from dental follicle stem cell to mitigate LPS-induced pulpitis in rats and facilitate pulp healing by promoting M2 macrophage polarization (Fig. 7A, C, and D) [74]. Functionally, DFSC-sEVs contained heat shock protein 70 (HSP70) that could be incorporated into lysosomes to safeguard their function and trigger mitophagy (Fig. 7B), which aided in the clearance of damaged mitochondria. This process primed inflammatory macrophages for oxidative phosphorylation, driving their transition towards M2 polarization.

### 4.2 Facilitating dental pulp regeneration or revascularization

Pulp regeneration aims to reconstruct a functional dentin-pulp complex complete with vasculature, nerves, and connective tissue [129,130]. The success of these strategies depends on stem cell survival, proliferation, differentiation, vasculogenesis, and neurogenesis, all of which are intimately linked to the bioenergetics and signaling functions of mitochondria [78].

Dental pulp revascularization is a regenerative strategy based on the principle of cell homing, primarily used to treat immature permanent teeth with necrotic pulp and periapical periodontitis [[131], [132], [133]]. This technique relies on the migration, proliferation, and differentiation of residual stem cells from the SCAP, accompanied by neovascularization and nerve ingrowth, ultimately leading to the formation of new vital tissue. This process occurs within the relatively hypoxic, inflammation-rich microenvironment of the root canal, posing severe challenges to cellular metabolic adaptability, particularly mitochondrial function [134]. Modulating SCAP mitochondrial function enhances cell survival rates and differentiation potential [79]. In addition, the type and functionality of tissue formed following current revascularization procedures are often unpredictable, frequently resulting in fibrous connective tissue or bone-like tissue rather than true pulp tissue [80]. Xuan's group demonstrated that apoptotic vesicles (apoVs) derived from human deciduous pulp stem cells (hDPSCs) promote angiogenesis and dental pulp regeneration by activating endothelial cells through the TFEB-autophagy pathway (Fig. 8 A). In a preclinical beagle dog model, apoVs successfully recruited endogenous endothelial cells and facilitated the formation of vascularized dental-pulp-like tissue (Fig. 8B and C). Moreover, the authors proved that the effect of apoVs was mediated by apoV-carried mitochondrial Tu translation elongation factor (TUFM, which is involved in translating mtDNA), further highlighting the role of mitochondria in revascularization (Fig. 8D–F) [75]. Future strategies may include using drugs or smart bioactive materials capable of precisely modulating the mitochondrial metabolic state of stem cells to guide their differentiation towards desired cell types (e.g., odontoblasts) [81].

### 4.3 Facilitating periapical bone tissue regeneration

Promoting the regeneration of periapical bone tissue is crucial for the long-term stability and function of the affected tooth in the management of periapical lesions [6]. Mitochondrial function plays an indispensable role in regulating the proliferation and differentiation of osteoprogenitor cells and osteoblasts during bone formation [82]. Studies indicate that as mesenchymal stem cells differentiate into osteoblasts, their metabolic pattern shifts from glycolysis to mitochondrial oxidative phosphorylation [83]. Zhang's group applied single-cell RNA sequencing to delineate the immune microenvironment associated with hydroxyapatite-driven alveolar bone regeneration, highlighting the pivotal role of macrophages in enhancing regenerative outcomes (Fig. 9A) [76]. Leveraging these insights, they engineered amorphous calcium zinc phosphate (ACZP) nanoparticles to serve as immunomodulatory agents. These ACZP nanoparticles were shown to expedite bone regeneration by promoting an anti-inflammatory macrophage phenotype, which involves restoring mitochondrial homeostasis and shifting macrophage metabolism toward oxidative phosphorylation (Fig. 9B–F), thus augmenting cellular energy dynamics.

*Opinion*: Existing endodontic materials such as MTA and Biodentine achieving great clinical repair, but they constrain true regeneration due to failed neurovascular integration. In addition, these materials exhibit limited anti-inflammatory capacity, relying on passive bacterial inhibition rather than active immunomodulation. Future strategies could explore metabolic modulatory interfaces—including regulation of mitochondrial redox homeostasis, thereby shifting the outcome from calcific repair to functional tissue regeneration. Furthermore, rather than loading materials with non-specific ROS scavengers (which abolish physiological mtROS signaling required for dentinogenesis), existing materials should be modified with mitophagy inducers (e.g., urolithin A or spermidine), which eliminates damaged mitochondria in the inflamed pulp while preserving healthy networks. From a clinical material modification perspective, while small-molecule modulators and exosomes demonstrate efficacy in preclinical models, their inactivation by strong alkaline conditions remains a concern. Future strategies may involve encapsulating exosomes in calcium alginate microspheres (buffering pH via Ca^2+^/OH^−^ interactions) and modifying small-molecule drugs with silane coupling agents to create prodrugs that hydrolyze upon return to physiological pH, slowly releasing active molecules. Building upon these insights, Table 4 further presents mitochondria-centered therapeutic concepts mapped to specific clinical scenarios.

**Table 4:** Types of mitochondrial stress and corresponding material design in different clinical scenarios.

Clinical classification [135]	Clinical scenarios [136,137]	Pathophysiological features & treatment goal [138]	Dominant type of mitochondrial stress [27] [139,140]	Material design focus/strategy [73,77,115]
Initial/Mild Pulpitis	Indirect/Direct pulp capping	∙ Hyperemia or localized mild coronal inflammation; ∙ Goal: Induce reparative dentin bridge formation to seal exposure and maintain pulp vitality.	∙ Mild oxidative stress; activation of protective mitophagy to maintain cellular homeostasis; transient increase in ATP demand.	Mild stimulation & Osteoinduction: Releasing optimal ions to maintain moderate ROS signaling for odontoblastic differentiation and dentin bridge formation.
Moderate pulpitis	Partial pulpotomy	∙ Extensive localized coronal inflammation; wound surface consists of stressed pulp tissue; ∙ Goal: Remove infected tissue and preserve remaining healthy pulp.	∙ Moderate-to-severe oxidative stress; mitophagy significantly upregulated but on the verge of decompensation; decreased membrane potential.	Apply carriers loaded with mitochondria-targeted agents to scavenge accumulated ROS and stabilize mitochondrial membrane potential.
Severe pulpitis	Full pulpotomy	∙ The infection has spread to the entire crown pulp, but the root pulp is healthy; ∙ Goal: Eliminate inflammation and promote pulp self-repair or achieve pulp regeneration.	∙ Severe network collapse & decompensation; evolution into destructive/excessive mitophagy; calcium overload; active mitochondria-mediated pyroptosis/apoptosis.	Apply carriers loaded with mitochondrial regulators to block destructive mitochondrial cascades.
Pulp necrosis	Regenerative endodontic procedures	∙ Pulp necrosis, empty root canal system; ∙ Goal: Achieve functional regeneration of pulp-dentin complex.	∙ Metabolic shock & severe hypoxia; insufficient mitochondrial biogenesis and extreme ATP depletion upon stem cell recruitment.	Scaffold-based metabolic rebooting. ∙For hypoxia: Employ collagen or silk fibroin scaffolds functionalized with oxygen-releasing materials. ∙ For paracrine signaling: Encapsulate mitochondrial biogenesis modulator in the scaffold.

Current mitochondria-interacting materials have demonstrated promising therapeutic potential in preclinical models, however, their passive and often non-specific modulation of mitochondria presents inherent limitations. The field is now transitioning toward more sophisticated strategies that transcend simple bioactivity to enable real-time monitoring, intelligent targeting, and precision engineering of mitochondria-safe interventions. The following section explores these forward-looking perspectives.

## 5 Opinion of pioneering endodontic materials from mitochondrial insights

Translating the profound insights of mitochondrial biology into innovative material design strategies for endodontics represents a pivotal driver for advancing the field. Specifically, next-generation endodontic materials centered on mitochondria can be pioneered via the following aspects: 1) mitochondrial monitoring; 2) mitochondrial assessment modalities; 3) precision mitochondrial target; 4) mitochondrial-safe material design; 5) promoting clinical transformation (Fig. 10).

### 5.1 Mitochondrial monitoring: a potential diagnostic tool for endodontics

Current pulp vitality assessment relies on subjective symptoms (e.g., pain response) or indirect imaging (e.g., periapical radiolucency), leading to a high rate of misdiagnosis of early-stage or partial necrosis (e.g., reversible pulpitis). However, mitochondrial dysfunction (such as membrane potential collapse or impaired respiratory chain function) precedes observable structural cellular damage. Real-time monitoring of mitochondrial parameters offers the potential to detect pulp pathologies earlier than conventional methods (such as thermal testing or X-rays), significantly enhancing diagnostic sensitivity. Recent advances in mitochondrial function monitoring illustrate this potential (Fig. 10A). For example, Ye et al. developed a mitochondria-specific blinking fluorescent bioprobe for nanoscopic monitoring of mitophagy [141]. Xiao's group developed a mitochondria-anchored ratiometric fluorescence thermometer (Mito-TEM 2.0) for real-time, accurate measurement of mitochondrial temperature based on the Förster resonance energy transfer, overcoming previous limitations and enabling breakthroughs in quantitative visualization of mitochondrial temperature changes during inflammatory processes [142]. Furthermore, a novel small molecule fluorescent probe UBER was developed for visualizing mtDNA base excision repair activity in live cells. UBER reacts with AP sites produced by BER, allowing for selective observation of mtDNA BER intermediates and offering a new method for studying mtDNA damage repair dynamics [143].

However, despite successful preclinical mitochondrial monitoring in other diseases and physiological contexts, effective monitoring under the unique structural and environmental conditions of dental pulp disease remains challenging. For instance, optical methods (such as near-infrared spectroscopy or optical coherence tomography) suffer significant signal attenuation when penetrating dense enamel, limiting deep-tissue probing capabilities [144]. An electrochemical monitoring strategy, integrating microelectrodes into a dental probe, may prove more suitable for direct post-access-cavity assessment (Fig. 10A). Beyond optical probes, developing alternative excitation probes capable of penetrating tooth structure, such as ultrasound or X-ray-based probes, could also address this limitation [145,146].

### 5.2 Mitochondrial assessment modalities

By integrating human intelligence with artificial intelligence (AI), brain-computer interfaces form a synergistic intelligent system that significantly enhances human performance. However, this technology often requires bulky wired instruments and significant invasiveness [147]. The oral cavity, with its unique open microenvironment rich in diagnostic biomarkers for disease diagnosis and its active organs such as teeth and tongue capable of transmitting signals, presents an opportunity for “oral-machine interfaces” to assist not only in endodontic diseases but also in a broader range of conditions (Fig. 10 B) [148]. Specifically, sensors integrated into dental appliances or orthodontic devices offer a convenient, non-invasive, and effective example of an oral-machine interface [147,149,150]. Future advancements may enable direct and indirect assessments of mitochondrial function by monitoring metabolic substances in saliva and exhaled gases, alongside physical and physiological indicators such as masticatory force, occlusal patterns, and lingual motility. Nevertheless, the development of mitochondrial assessment modalities must navigate the intricate challenges posed by the oral microenvironment's numerous interfering factors and the enduring need for a sustainable power supply to these sensors.

### 5.3 Enhancing targeting specificity to mitochondria

Mitochondrial targeting not only improves therapeutic efficacy but also minimizes off-target effects on healthy cells or non-target organelles, representing a cornerstone for developing high-precision endodontic therapeutics (Fig. 10C).

#### 5.3.1 Chemical modification strategies

Mitochondrial membrane potential dissipation is frequently equated with the point of irreversible commitment to cell death. While modest and early depolarization remains reversible, mitochondrial outer membrane permeabilization drives the release of cytochrome *c*, thereby triggering an irreversible proteolytic cascade. Mitochondrial membrane potential-sensing delivery systems should be developed to selectively target this pre-death population, maximizing pharmacodynamic precision and minimizing off-target toxicity, albeit with temporal parameters and efficacy thresholds that vary significantly across cellular contexts.

Exploiting mitochondrial membrane potential (negative membrane potential) or transporter proteins constitutes a classical targeting approach [139,140]. Covalent conjugation of mitochondria-targeting moieties (e.g., triphenylphosphonium cation) or de novo quaternary ammonium salts to bioactive molecules or nanocarriers enables efficient delivery of cargos to the mitochondria [151,152]. However, triphenylphosphonium cation-based materials rely on mitochondrial membrane potential; a reduction in mitochondrial membrane potential can severely impact mitochondrial targeting efficiency. Hong et al. developed a mitochondrial probe that combines the cationic quinolinium's mitochondrial targeting capability with the C_12_ chain's ability to embed in the mitochondrial membrane [153]. The membrane-binding property of C_12_ enables firm attachment to mitochondria, ensuring that the probe can target mitochondria without relying on mitochondrial membrane potential.

#### 5.3.2 Bio-recognition strategies: dual cell-organelle targeting paradigms

Conventional nanocarriers often face off-target effects in complex tissue microenvironments. In the intricate context of dental pulp regeneration scenarios, precisely regulating the mitochondrial dynamics across diverse cell types holds significant importance. To address this challenge, dual “cell-organelle” targeting systems can be designed by synergistically integrating cell surface biomarker recognition with mitochondrial targeting sequences [154]. Nevertheless, this approach is technically more challenging, requiring solutions to obstacles such as steric hindrance of targeting ligands and low transmembrane translocation efficiency.

### 5.4 Reduce toxicity to mitochondria

Traditional and some novel pulp treatment materials often interfere with mitochondrial function due to cytotoxicity from components or degradation products, leading to cellular metabolic disorders or apoptosis [51,53,54,155,156]. The development of next-generation bioactive materials must adhere to the “mitochondria-friendly” principle, balancing therapeutic efficacy and mitochondrial safety through a tripartite strategy involving source component screening, release kinetics modulation, and functional toxicity evaluation (Fig. 10D). Future materials should prioritize biocompatible and metabolically inert raw materials and integrate mitochondrial toxicity into early screening criteria, establishing rapid pre-screening platforms based on in vitro mitochondrial toxicity detection models. Optimal ion or signaling molecule concentrations promote mitochondrial function and cell differentiation, while excessively high concentrations or rapid release rates cause mitochondrial Ca^2+^ overload, ROS bursts, or metabolic dysregulation. Precise structural design (e.g., porosity, crystallinity) and chemical composition are required to achieve slow, controlled, and sustained release, maintaining concentrations within the “therapeutic window” of mitochondrial tolerance and functional benefit.

### 5.5 Promote clinical transformaton

Clinical translation of bioactive materials represents a critical bottleneck in endodontic regeneration (Fig. 10E). Although large animal models (e.g., porcine) better mimic human dental physiology regarding pulp chamber dimensions and tissue healing kinetics, their utilization is frequently hindered by stringent ethical regulations and substantial financial investments [157]. Conversely, while small rodents offer advantages for experimental manipulation, significant discrepancies exist in dental architecture and oral microenvironment compared to humans [158]. Notably, current pulp exposure protocols in these models fail to adequately replicate the chronic inflammatory progression and periapical pathogenesis characteristic of clinical pulp diseases [159]. In this context, organoid cultures and organ-on-a-chip platforms have emerged as promising alternatives, enabling precise control of cellular microenvironments while circumventing ethical constraints associated with in vivo experimentation [160]. Nevertheless, these sophisticated systems currently face inherent technical limitations, including incomplete vascularization, absent neural innervation, and oversimplified immune microenvironments, which may compromise their translational predictability for clinical outcomes.

From a regulatory perspective, most countries adopt a conservative stance toward the development of new endodontic materials. Pulp capping materials containing pharmaceutical ingredients are typically classified as high-risk Class III medical devices, necessitating extensive preclinical and clinical testing before market approval [161]. Consequently, compared with other medical disciplines, endodontic materials remain relatively conventional, lacking smart functionalities and targeted delivery capabilities [160]. Therefore, rather than developing entirely new materials devoid of clinical history, we propose modifying existing endodontic materials by incorporating small-molecule drugs or bioactive factors. This approach allows researchers to evaluate the modified bioactive material as a substantial modification rather than as a novel entity, thereby shortening the translation timeline and reducing development costs [162]. Additionally, repurposing drugs or small molecules already approved in other medical fields for dental applications could further facilitate clinical translation [163]. Government attitudes toward new endodontic materials vary considerably across countries. While regulatory bodies in Europe and North America remain cautious, China has adopted a more proactive policy toward biomedical materials, often providing favorable testing environments and financial incentives for innovators [164]. As a result, the average time required to obtain Class III device registration in China is approximately shorter than in Western countries [165], and domestic dental materials are projected to capture over 50% of the Chinese market. However, Chinese manufacturers face significant challenges in international markets, particularly in accessing premium segments in Europe and North America, due to insufficient clinical evidence and differences in evaluation standards. Future efforts should focus on aligning with international standards and actively participating in standard-setting processes to ensure global competitiveness.

## 6 Conclusions

This leading opinion paper systematically explores the role of mitochondrial biology in elucidating the mechanisms of endodontic diseases, while emphasizing how these insights can guide the development of next-generation endodontic bioactive materials. Accordingly, we propose a conceptual framework for future mitochondria-directed precision endodontics (not yet clinically established). In the future, next-generation pulp therapeutic materials with clearer mechanisms and superior efficacy can be anticipated, drived by enhanced mitochondrial targeting specificity, minimized potential mitochondrial toxicity, and advanced mitochondrial assessment modalities. Finally, bridging the gap between promising preclinical findings and widespread clinical translation remains a major hurdle for any novel bioactive material. This endeavor demands innovation not just in material science, but also in manufacturing technologies, preclinical modeling, and clinical trial design.

## Ethics approval and consent to participate

Not applicable.

## CRediT authorship contribution statement

**Shi Cheng:** Data curation, Writing – original draft, Writing – review & editing. **Xin-Ya Liu:** Data curation, Writing – original draft, Writing – review & editing. **Lu Zhou:** Conceptualization, Writing – original draft, Writing – review & editing. **Han-Qing Mao:** Formal analysis, Investigation. **Yuan-Hao Wen:** Formal analysis, Investigation. **Xiang Meng:** Data curation, Formal analysis. **Lu Zhang:** Conceptualization, Supervision, Validation. **Zhi Chen:** Conceptualization, Supervision, Validation.

## Declaration of competing interest

No potential conflict of interest was reported by the authors.

## Data Availability

No data was used for the research described in the article.

## Abbreviations

DPSCs: dental pulp stem cells
DAMP: damage-associated molecular pattern
SCAPs: apical papilla stem cells
TCA: tricarboxylic acid
TFAM: mitochondrial transcription factor A
mtROS: mitochondrial reactive oxygen species
LPS: lipopolysaccharide
PAMPs: pathogen-associated molecularpatterns
LTA: lipoteichoic acid
MTA: mineral trioxide aggregate
ZOE: zinc oxide eugenol
DFSC: dental follicle stem cell
VPT: vital pulp therapy
apoVs: apoptotic vesicles
hDPSCs: human deciduous pulp stem cells
TUFM: mitochondrial Tu translation elongation factor
ACZP: amorphous calcium zinc phosphate
AI: artificial intelligence.
