# Supplementary material for: Pioneering next-generation bioactive materials for endodontics: Insights of mitochondrial biology
Source: Bioact Mater. 2026 Jun 4;65:292–310. doi: 10.1016/j.bioactmat.2026.05.040 (PMC13265711; doi:10.1016/j.bioactmat.2026.05.040)
Supplement: Multimedia component 1 [file mmc1.docx]

**Table S1.** Mitochondria-mediated different cell death modes.

| **Features** | **Trigger Factors** | **Core Events** | **Key Regulators** | **Morphological Features** | **Inhibitors** |
| --- | --- | --- | --- | --- | --- |
| **Apoptosis** | Death receptor ligation | Caspase cascade activation | Caspase-3/8/9;  Bcl-2 family | Shrinkage/apoptotic bodies | Z-VAD-FMK;  Q-VD-OPh |
| **Necrosis** | Hypoxia/toxins/ Physical damage | ATP depletion/  membrane rupture | No specific molecules | Swelling/organelle dissolution | / |
| **Necroptosis** | TNF-R1/ZBP1 activation | RIPK1/RIPK3/MLKL phosphorylation | RIPK1/RIPK3/MLKL | Plasma membrane rupture | Necrostatin-1;  GSK872 |
| **Pyroptosis** | PAMPs recognition | Gasdermin pore formation | Caspase-1/4/5/11  Gasdermins | Pyroptotic body  formation | VX-765  Disulfiram |
| **NETosis** | ROS/Ca^2+^ influx | Chromatin decondensation | PAD4/MPO | NETs extrusion | Cl-amidine;  DNase I |
| **Ferroptosis** | Fe²⁺ overload/  GPX4 inhibition | Lipid peroxide  accumulation | GPX4/ACSL4/FSP1 | Mitochondrial cristae loss | Ferrostatin-1;  Liproxstatin-1 |
| **Cuproptosis** | Cu²⁺ overload | FDX1activation/  Cu-protein aggregation | FDX1/DLAT/LIAS | Mitochondrial swelling | Tetrathiomolybdate |
